# Integration of Specialist Palliative Care into Tertiary Hospitals: A Multicenter Point Prevalence Survey from Thailand

**DOI:** 10.1089/pmr.2021.0003

**Published:** 2021-10-06

**Authors:** Srivieng Pairojkul, Rojanasak Thongkhamcharoen, Attakorn Raksasataya, Chalermsri Sorasit, Pakkawee Nakawiro, Supannee Sudsa, Chaleow Sattamai, Napassawan Puripanpinyo, Nittha Oerareemitr, Boriboon Raksadaen, Patthamaporn Apaijitt, Busaya Santisant, Pruksaporn Thammachote, Sermsuk Thunyawan, Valika Rattanachun, Vittawin Fagcharoenpol

**Affiliations:** ^1^ Karunruk Palliative Care Center, Faculty of Medicine, Khon Kaen University, Khon Kaen, Thailand.; ^2^ President, Thai Palliative Care Society, Khon Kaen, Thailand.; ^3^ Department of Social Medicine, Maesot General Hospital, Tak, Thailand.; ^4^ Department of Social Medicine, Phra Nakhon Si Ayutthaya Regional Hospital, Phra Nakhon Si Ayutthaya, Thailand.; ^5^ Department of Medicine, Udonthani Regional Hospital, Udonthani, Thailand.; ^6^ Department of Palliative Care and Long Term Care, Surin Regional Hospital, Surin, Thailand.; ^7^ Department of Social Medicine, Roi-Et Regional Hospital, Roi-Et, Thailand.; ^8^ Department of Pulmonary and Critical Care Medicine, Faculty of Medicine, Phramongkutklao Hospital, Bangkok, Thailand.; ^9^ Department of Social Medicine, Buddhachinaraj Regional Hospital, Phitsanulok, Thailand.; ^10^ Department of Nursing, Nangrong General Hospital, Buriram, Thailand.; ^11^ Jairak Palliative Care Center, Vachira Phuket Regional Hospital, Phuket, Thailand.; ^12^ Department of Social Medicine, Songkhla General Hospital, Songkhla, Thailand.; ^13^ Department of Nursing, Loei General Hospital, Loei, Thailand.; ^14^ Department of Social Medicine, Nan General Hospital, Nan, Thailand.; ^15^ Department of Social Medicine, Buriram Regional Hospital, Buriram, Thailand.

**Keywords:** accessibility, advance care planning, palliative care specialist, prevalence, strong opioids prescription, Thailand

## Abstract

***Background:*** Accessibility and quality of hospital-based palliative care in Thailand have received scant attention.

***Objective:*** To determine the prevalence of inpatients who require in-hospital palliative care, to identify the proportion with access to specialist palliative care, and to define the factors associated with accessibility to specialist palliative care.

***Design:*** A cross-sectional analysis of a multicenter survey.

***Setting/Subjects:*** We surveyed all hospitalized patients from Thailand's four regions admitted to 14 tertiary care hospitals.

***Measurements:*** We used the Supportive and Palliative Care Indicators Tool to identify palliative care patients then reviewed their medical records. We categorized hospitalized palliative care patients into a palliative care consultation group and a nonconsultation group. The odds ratio (OR) between patient characteristics and patient groups was estimated using binary logistic regression.

***Results:*** One-fifth (18.7%) of hospitalized patients were palliative care patients, whereas only 17.3% received a specialist palliative care consult. Of these, one-third (28.4%) received advance care planning (ACP) documentation. One-quarter of patients in pain were not prescribed analgesics. The logistic regression analysis revealed that palliative care consultations were associated with patients >65 years (OR = 1.830, 95% confidence interval [CI]: 1.122−2.987), a cancer diagnosis (OR = 2.640, 95% CI: 1.478−4.718), strong opioids prescription (OR = 5.519, 95% CI: 3.217−9.469), and ACP documentation (OR = 50.149, 95% CI: 28.239−89.059).

***Conclusions:*** The prevalence of hospitalized palliative care patients in Thailand is comparable with that in developed countries; however, accessibility remains a significant gap, as specialist palliative care is associated with the quality of palliative care service.

## Introduction

The World Health Organization estimated that globally only 14% of people who need palliative care have access to it.^1^ The main barriers include lack of national health policies and system support for palliative care, inadequate training for health professionals, and limited opioids access.^2^ A previous survey showed differences in the development level of specialist palliative care services in the Asia-Pacific region.^3^ In 2012, Thailand was categorized as having “Isolated palliative care activity” (level 3a).^4^ In 2017, a new survey categorized Thailand as having “Palliative care at preliminary stage of integration” (level 4a).^5^ Palliative care service in Thailand needs improvement before being able to be categorized as “Palliative care at advanced stage of integration” (level 4b).

Thailand now has a sizeable elderly population >60 years.^6^ Cancer, strokes, and coronary heart disease have become leading causes of death just as they are in developed countries.^7^ Systematic palliative care in Thailand has been developing for two decades. In the past, oral morphine, palliative care personnel, and their services were less available in Thai hospitals.^8,9^ Thailand was 44th in the world ranking and 10th in the Asia-Pacific region in “The 2015 quality of death index.”^10^ With the support and collaboration of multiple organizations from different sectors, palliative care development has markedly progressed for the past 10 years.^11^ Thailand passed Section 12 of the National Health Act, B.E. 2550 (2007), endorsing the right of a terminally ill patient to refuse futile medical interventions by written advance directives.^12^

The Thai government then issued a national policy for long-term care and palliative care systems in 2014.^12^ One target was to establish palliative care units in every hospital by 2016.^11,12^ A specialist palliative care unit provides inpatient consultation service and an outpatient clinic. These units coordinate with the primary care services and facilitate patient referral to the appropriate community health care networks.

Almost 85% of government hospitals had a palliative care committee, and many (58%) of those had access to strong oral opioids.^13^ The Ministry of Public Health (MOPH) established standard palliative guidelines and an essential palliative care drugs list for MOPH hospitals.^13,14^ The MOPH has set the Key Performance Indicators that track the overall quality of palliative care service in the country, including the rate of opioid coverage in palliative care patients, and the rate of palliative care patients receiving advance care planning (ACP) process.^15^ Forty-five percent of the total national deaths occur in hospitals^7^; however, the national policy does not involve the provision of palliative care wards or hospices.^9^ The quality of an inpatient palliative care service model without a hospice needs investigation.

The Thai Palliative Care Network (TPCN)—a group of well-trained palliative care personnel working in established palliative care centers across Thailand—undertook a point prevalence survey to (1) determine the prevalence of inpatients who need palliative care in hospital, (2) identify the proportion of inpatient-palliative patients who have access to specialist palliative care, and (3) define the factors associated with the accessibility of specialist palliative care.

## Methods

### Study design

A cross-sectional contemporaneous multicenter point prevalence design was selected to estimate the number of palliative care patients in tertiary care hospitals. The study protocol was approved by the ethics committee of Khon Kaen University, Thailand (Reference number: HE 611525).

### Setting

Fourteen tertiary care hospitals from the four regions of Thailand were purposely sampled. There were two university hospitals, seven regional hospitals, and five general hospitals participating in the research. Regional hospitals are designated as supra-tertiary care, acting as a referral center for general and community hospitals. General hospitals are smaller tertiary care centers that are provincial referral centers for community hospitals. These hospitals have specialist palliative care consultation teams. The studied hospitals are located in the northeast region (one university hospital, four regional hospitals, and two general hospitals), the northern region (one regional hospital and two general hospitals), the central region (one university hospital and one regional hospital), and the southern region (one regional hospital and one general hospital). The Karunruk Palliative Care Center, the university palliative care center in the northeast region, was the research focal point for the study.^16^

### Eligibility criteria

All patients who were inpatients between November 26 and 28, 2018 were enrolled into the study. Pediatric postpartum psychiatric admissions and the patients who were admitted on the date of data collection were excluded.

Supportive and Palliative Care Indicators Tool (SPICT™) version 2017 was used to identify palliative patients who might benefit from palliative care.^17^ To meet the SPICT criteria, we needed at least one indicator of the deteriorating health and at least one indicator of the advanced condition. All researchers (the palliative care nurses or the palliative care doctors) had been trained through teleconference how to interpret the SPICT criteria and to understand the research protocol. Hospitalized patients were reviewed with respect to their inpatient chart records (ICR) and electronic medical records (EMR) using SPICT version 17.

### Measurements

Information retrieved from the EMR included demographics, diagnosis, comorbidities, the length of stay (LOS; the number of days from hospital admission to the date of data collection), unplanned hospital visits (the number of unplanned emergency room (ER) visits and unplanned admissions six months before admission), and comorbidities. Information reviewed from the ICR included treatments [endotracheal (ET) intubation, mechanical ventilation, inotropic drug administration, antibiotics, nasogastric (NG) tube feeding, parenteral nutrition, chemotherapy (CMT), palliative radiotherapy (RT), palliative surgery, and symptomatic management], conscious level, performance status, symptom burden, pain intensity record, analgesic prescription, specialist palliative care consultation status, and ACP status.

### Data analysis

SPSS for Windows version 16 was used for the statistical analyses. Categorical variables were presented as frequencies and percentages. Parametric data were described as means and standard deviations (SDs). Nonparametric data were presented as medians and interquartile ranges (IQRs). We categorized palliative care patients into two groups: a palliative consultation group and a nonconsultation group. The odds ratios (ORs) and 95% confidence intervals (95% CIs) between independent variables and patient groups were estimated using binary logistic regression. A two-tailed test, *p*-value <0.05 was considered significant. The independent variables included age, diagnosis, cognitive level, performance status, pain intensity record, analgesic prescription, ACP status, and ET intubation.

## Results

After pediatric, postpartum, and psychiatric admissions were excluded, 5763 hospitalized patients were surveyed. Table 1 presents the characteristics of palliative care patients according to the SPICT criteria. The prevalence of palliative care patients was 18.7% (1079 cases), ranging between 12% and 35% among the participating hospitals. Palliative care specialists were consulted in only 187 cases (17.3%). The mean age was 62.8 (16.4 SD) years, and 97.7% of these patients had health care coverage. Two-thirds (62.4%) of the studied population lived in the northeast region of Thailand. Patients were admitted to university hospitals (19.6%), regional hospitals (58.5%), and general hospitals (21.9%). The highest palliative inpatient burden was found in the medical ward (59.2%). At least one comorbidity was found in 68% of palliative care patients. The median number of patients with two comorbidities was 20.4%, and only one-third had no comorbidity. Most (81.6%) had unplanned hospital visits with a median IQR of 6 (range 2–10) visits. The median LOS IQR was 7 (range 3–16) days. ACP was in place in 28.4%. The performance status of nearly half of these patients (48.2%) was bedridden and while nearly one-fifth (17.6%) were confined to bed for >50% of the time. Only one-third (34.2%) of palliative care patients were self-dependent. The overall cognitive level of patients was 69.6% fully conscious, 23.7% impaired mental status, and 6.4% in coma. The burden of symptoms included pain (49.6%), dyspnea (45.7%), gastrointestinal (GI) symptoms (33.8%), and/or other symptoms (25.5%). Life-sustaining treatments included intubation (24.6%), ventilator (22.4%), and inotropic drug administration (7.9%). Antibiotics were prescribed in 60.9%. NG tube feeding and parenteral nutrition were given to 34.6% and 15.4% of these patients, respectively. Other treatments included CMT (20.4%), palliative RT (7.5%), and palliative surgery (9.5%). Missing data were found in symptom burden [dyspnea 3.2% (35 cases), pain 4.9% (53 cases), GI symptom 7.6% (82 cases), and other symptoms 15.8% (171 cases)] and pain intensity record 4.4% (47 cases)]. We presented the missing data as “not assessed.”

**Table 1.** Characteristics of Palliative Care Patients According to Supportive and Palliative Care Indicators Tool (*N* = 1079)

Characteristics	*n* (%)	Characteristics	*n* (%)
Age, years (mean age ± SD)	62.76 ± 16.4	Specialist palliative care consultation	187 (17.3)
≤65	607 (56.3)	Advance care planning status	306 (28.4)
>65	472 (43.7)	LOS (days) median (IQR)	7 (3–16)
Cancer diagnosis		Unplanned visit	
Cancer	541 (50.1)	Number of cases (%)	880 (81.6)
Noncancer	538 (49.9)	Median (IQR), visits	6 (2–10)
Living region		Gender	
Northern	149 (13.8)	Male	585 (54.2)
Northeastern	673 (62.4)	Female	494 (45.8)
Central	150 (13.9)	Treatment	
South	107 (9.9)	Endotracheal intubation	265 (24.6)
Hospital types		ventilator	242 (22.4)
University hospital	212 (19.6)	Inotropic drug administration	85 (7.9)
Regional hospital	631 (58.5)	Antibiotics	657 (60.9)
General hospital	236 (21.9)	Nasogastric tube feeding	373 (34.6)
		Parenteral nutrition	166 (15.4)
Number of chronic conditions		Chemotherapy	220 (20.4)
0	346 (32.1)	Palliative radiotherapy	81 (7.5)
1	347 (32.2)	Palliative surgery	102 (9.5)
2	220 (20.4)	Cognitive level	
≥3	166 (15.4)	Normal	751 (69.6)
		Impair	256 (23.7)
		Coma	72 (6.4)
Department		Performance status	
Medical ward	639 (59.2)	Full function	116 (10.8)
Surgical ward	269 (24.9)	Light activity	186 (17.2)
Gynecological ward	40 (3.7)	<50% in bed	67 (6.2)
Orthopedic ward	33 (3.1)	>50% in bed	190 (17.6)
Others	98 (9.1)	Bedridden	520 (48.2)
Health care insurance		Symptom burden	
Civil servant medical benefit	267 (24.7)	Pain	535 (49.6)
Universal coverage scheme	717 (66.5)	Dyspnea	493 (45.7)
Social security scheme	70 (6.5)	Gastrointestinal symptoms	365 (33.8)
Self-payment	25 (2.3)	Others	275 (25.5)

*n* (%), case number (%); IQR, interquartile range; LOS, length of stay; SD, standard deviation.

**FIG. 1.**
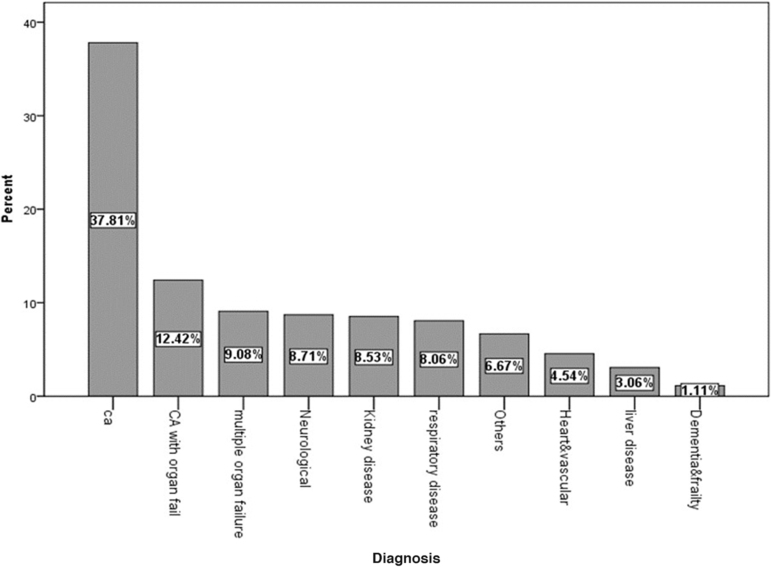
Main diagnoses of palliative care patients according to Supportive and Palliative Care Indicators Tool version 17. CA, cancer.

The diagnoses % of the palliative care patients were cancer in 37.8%, cancer with organ failure in 12.4%, and noncancer in 49.9 (Fig. 1). The primary sites of cancer were hepatobiliary (23.0%), GI (22.4%), head and neck (15.9%), hematologic (11.8%), lung (10.53%), and others (12.0%).

Pain assessments were documented in 95.6% of palliative care inpatients and 49.6% of the palliative inpatients had pain. We found that in 24.1% of patients suffering from pain, analgesics were not prescribed. Moreover, strong opioids were prescribed for only 48% of patients in pain (Table 2).

**Table 2.** The Proportion of Pain Symptom and Analgesic Prescription in Palliative Care Patients

Characteristics	Total, *n* (%)	Pain, *n* (%)	No pain, *n* (%)	Not assessed, *n* (%)
Pain symptom record	1079 (100)	545 (50.5)	487 (45.1)	47 (4.4)
Analgesic prescription				
No analgesic	561 (52)	129 (24.1)	393 (80.0)	39 (73.6)
Nonopioids	144 (13.3)	84 (15.7)	54 (11.0)	6 (11.3)
Weak opioids	79 (7.3)	65 (12.1)	13 (2.6)	1 (1.9)
Strong opioids	295 (27.3)	257 (48.0)	31 (6.3)	7 (13.2)

*n* (%), case number (%).

Table 3 shows the patient variables and their bivariate associations with specialist palliative care consultation status. We recategorized cognitive level into normal and impaired (impaired plus coma conscious level). We also regrouped performance status from five groups to two groups (Table 1); nonbedridden (normal, light activity, <50% and >50% in bed) and bedridden. The proportion of these variables by specialist palliative consultation groups are presented—the comparison being between consult versus not consult.

**Table 3.** Patients' Variables and Their Association with Specialist Palliative Care Consultation Status

Characteristics	Specialist palliative consultation status		95% CI for OR			
	Not consult (*N* = 892)	Consult (*N* = 187)				
	*n* (%)	*n* (%)	OR	Lower	Upper	*p*
Age group, years			1.830	1.122	2.987	0.016
≤65 (Ref.)	514 (57.6)	93 (49.7)				
>65	378 (42.4)	94 (50.3)				
Cancer diagnosis			2.640	1.478	4.718	0.001
Noncancer (Ref.)	474 (53.1)	64 (34.2)				
Cancer	418 (46.9)	123 (65.8)				
Cognitive level			1.783	0.958	3.318	0.068
Normal (Ref.)	640 (71.7)	111 (59.4)				
Impaired to coma	252 (28.3)	76 (40.6)				
Performance Status			0.986	0.548	1.773	0.963
Nonbedridden (Ref.)	484 (54.3)	75 (40.1)				
Bedridden	408 (45.7)	112 (59.9)				
Advance care planning			50.149	28.239	89.059	<0.001
No (Ref.)	752 (84.3)	21 (11.2)				
Yes	140 (15.7)	166 (88.8)				
Pain intensity record			0.880	0.513	1.509	0.642
No (Ref.)	434 (50.7)	53 (30.1)				
Yes	422 (49.3)	123 (69.9)				
Analgesic prescription			5.519	3.217	9.469	<0.001
No strong opioids (Ref.)	712 (79.8)	72 (38.5)				
Strong opioids	180 (20.2)	115 (61.5)				
ET intubation			0.949	0.526	1.712	0.861
No (Ref.)	683 (76.6)	131 (70.1)				
Yes	209 (23.4)	56 (29.9)				

ET, endotracheal.

In the binary logistic regression analysis, individuals >65 years were more likely to receive a specialist palliative care consultation (OR = 1.830, 95% CI: 1.122–2.987) than younger patients. Cancer patients were 2.6 times more likely to get a specialist palliative care consultation than noncancer patients (95% CI: 1.5–4.7 times). Strong opioids prescription and ACP documentation were associated with a specialist palliative care consultation (OR = 5.5, 95% CI: 3.2–9.5 and OR = 50.1, 95% CI: 28.2–89.1, respectively). Specialist palliative care consultation was not significantly associated with performance status, level of consciousness, pain intensity record, and ET intubation.

## Discussion

Our research documents the large unmet need in Thai tertiary hospitals for the provision of palliative care services. The prevalence of palliative care patients in the survey is comparable with similar studies.^18–22^ The accessibility of specialist palliative care in this study (17.3%) was higher than another similar study in Thailand (6.1%)^23^; however, the proportion of palliative care patients with access to specialist palliative care was still lower than that in developed countries (25%–38%).^18,22,24^ Noncancer patients and younger age groups are less likely to get specialist palliative care services. This study showed that the characteristics of palliative care patients were frail, debilitated, and suffered from multiple symptoms. They have a high proportion and frequency of unplanned hospital visits, prolonged LOS and a high incidence of receiving invasive treatment/procedures. Grim et al.^25^ and Nipp et al.^26^ described the phenomenon of uncontrolled symptoms and complicated progression of diseases leading to unplanned revisits.

Although annual morphine equivalence consumption in Thailand rose from 3.96 mg per capita in 2010 to 5.85 mg per capita in 2015, the highest in Southeast Asia, it is far from the optimal level of usage (i.e., compared with the global average of 61.5 mg per capita).^27^ We found that pain was presented in 50% of palliative care patients and only 48% received strong opioids. Our findings highlight the inadequate implementation of a strong opioids availability policy. Lack of training, periodic shortages of opioids, and negative attitudes toward strong opioids are examples of barriers to the use of opioids for cancer pain management in Thailand.^28,29^

Regarding ACP, we found that a consultation group had more ACP documentation than a nonconsultation group, whereas the rate of ET intubation was not significantly different. Enguidanos et al.^30^ reported that late ACP documentation in the last months of life are associated with higher rates of preferences toward aggressive care. Thai tertiary care, hospital specialist, and palliative care teams are so few in number and have limited resources so control of symptoms and ACP cannot always be addressed. Furthermore, confined patient physical status and limited unresponsive mental status may interfere with ACP. In a systematic review, Zwakman et al.^31^ found that palliative care patients are ambivalent to discussing ACP, underscoring that patient readiness and openness are axiomatic to the ACP process. Relatedly, family members also strongly influence patient end-of-life decision making; particularly in Asian culture where the extended family continues to play a significant role in age care.^32^

To improve accessibility and quality of palliative care service for tertiary care hospitals in Thailand, all health care staff in all inpatient care services should be prepared to perform timely specialist palliative consultations.^1^ Thongkhamcharoen et al.^33^ surveyed the regulation of opioids in Thai government hospitals and found that physicians are the gatekeepers for patient access to opioids. Specialist palliative care teams should thus coordinate with primary physicians to achieve the best quality of care for both palliative care patients and their families.^29,34^ To improve accessibility to palliative care, appropriate palliative care screening tools should be implemented early for hospitalized palliative patients. According to the national policy movement, the expert taskforce for ACP, under the supervision of Nation Health Commission Office Thailand, has been working on the definitions and guidelines for ACP tailored for the Thai context.^35^

A better financial model to support inpatient palliative care model would represent another strategy for improving the quality of palliative care service, but Thailand has a limited number of financial subsidies.^10^ Although the trend of in-hospital death is increasing in Thailand, financial support from the national funding agency for palliative care is limited to reimbursement for home care.^36^ The results of this study suggest there is a cost to the health care system when palliative care patients do not receive care from specialist palliative care service. The costs are in inadequate pain management, frequent unplanned hospital visits, prolonged LOS, and high incidence of invasive procedures in palliative care patients. Smith et al. provided evidence that planned palliative care is correlated with treatment cost savings.^37^ In this regard, The Economist Intelligence Unit reported that palliative care can reduce the burden on health care systems and limit the use of costly but futile treatments.^10^

The limitation of this study is that it was a retrospective document review. As such, the quality of medical records could possibly have an impact on the results. The centers that were recruited into the survey are the principal palliative care centers in Thailand, but if the survey had included other hospitals where even less palliative care is offered, the prevalence of palliative care service may have been even lower. In addition, the study only recruited government hospitals, so the study does not represent palliative care in the private hospital setting. There is also need of further studies to evaluate the prevalence of the palliative care patients in the community.

## Conclusions

The prevalence of hospitalized palliative care patients in Thailand is comparable with that in developed countries. Palliative inpatients have long hospital stays, frequent unplanned visits, more invasive procedures, and a high symptom burden regardless of diagnosis. Pain management is the primary area needing improvement. Specialist palliative care is associated with quality of palliative care service; however, only a small proportion of palliative care patients have access to specialist palliative care services. Noncancer palliative care patients are less likely to access palliative care services than cancer patients. Poor accessibility, late consultation, and a high proportion of palliative care patients receiving invasive procedures are possibly a reflection of a lack of awareness and palliative care education among health professionals.

## Abbreviations Used

ACP: advance care planning
CA: cancer
CI: confidence interval
CMT: chemotherapy
EMR: electronic medical record
ET: endotracheal
GI: gastrointestinal
ICR: inpatient chart records
IQR: interquartile range
LOS: length of stay
MOPH: Ministry of Public Health
NG: nasogastric
OR: odds ratio
RT: radiotherapy
SD: standard deviation
SPICT: Supportive and Palliative Care Indicators Tool
TPCN: Thai Palliative Care Network
